# Theory of sampling and its application in tissue based diagnosis

**DOI:** 10.1186/1746-1596-4-6

**Published:** 2009-02-16

**Authors:** Klaus Kayser, Holger Schultz, Torsten Goldmann, Jürgen Görtler, Gian Kayser, Ekkehard Vollmer

**Affiliations:** 1 UICC-TPCC, Institute of Pathology, Charite, Berlin, Germany; 2 Clin. & Exp. Pathology, Research Center Borstel, Borstel, Germany; 3 Deep Computing, IBM, Amsterdam, the Netherlands; 4 Institute of Pathology, University of Freiburg, Freiburg, Germany

## Abstract

**Background:**

A general theory of sampling and its application in tissue based diagnosis is presented. Sampling is defined as extraction of information from certain limited spaces and its transformation into a statement or measure that is valid for the entire (reference) space. The procedure should be reproducible in time and space, i.e. give the same results when applied under similar circumstances. Sampling includes two different aspects, the procedure of sample selection and the efficiency of its performance. The practical performance of sample selection focuses on search for localization of specific compartments within the basic space, and search for presence of specific compartments.

**Methods:**

When a sampling procedure is applied in diagnostic processes two different procedures can be distinguished: I) the evaluation of a diagnostic significance of a certain object, which is the probability that the object can be grouped into a certain diagnosis, and II) the probability to detect these basic units. Sampling can be performed without or with external knowledge, such as size of searched objects, neighbourhood conditions, spatial distribution of objects, etc. If the sample size is much larger than the object size, the application of a translation invariant transformation results in Kriege's formula, which is widely used in search for ores. Usually, sampling is performed in a series of area (space) selections of identical size. The size can be defined in relation to the reference space or according to interspatial relationship. The first method is called random sampling, the second stratified sampling.

**Results:**

Random sampling does not require knowledge about the reference space, and is used to estimate the number and size of objects. Estimated features include area (volume) fraction, numerical, boundary and surface densities. Stratified sampling requires the knowledge of objects (and their features) and evaluates spatial features in relation to the detected objects (for example grey value distribution around an object). It serves also for the definition of parameters of the probability function in so – called active segmentation.

**Conclusion:**

The method is useful in standardization of images derived from immunohistochemically stained slides, and implemented in the EAMUS™ system . It can also be applied for the search of "objects possessing an amplification function", i.e. a rare event with "steering function". A formula to calculate the efficiency and potential error rate of the described sampling procedures is given.

## Introduction

Diagnostic surgical pathology or tissue – based diagnosis is confronted with remarkable changes in its environment and workflow. The technological progress has led to a broad application of molecular biological methods such as Fluorescent in Situ Hybridization (FISH), and other DNA – sequence amplification techniques [1,2]. Commercially available slide scanners digitize a complete glass slide within a few minutes, and permit the implementation of completely digitized images into routine diagnostics [3,4]. In other words, the workload of a pathologist increases steadily not only by increase of material, but, in addition, due to the mandatory introduction of new, still tissue – based diagnostic technologies. Thus, the question arises: How can the availability of and access to digitized histological slides (virtual slides) be used to release the diagnostic pathologist from time consuming work steps in order to make the pathologist's work more effective and disease related?

In the early days of telepathology, which can be considered to be the "mother of the digital pathologist's world", several authors reported on the diagnostic accuracy of viewing digitized slides in comparison to conventional microscopy [4-8]. The results were clear: the diagnostic accuracy viewing at a digitized (or virtual) slide is indistinguishable to that of conventional microscopy; however, the required time is essentially longer [9,10]. The non appropriate and more time consuming search for appropriate fields of view or the performed sampling procedure are obviously one reason of these constraints. To our knowledge, the theory of sampling in cytology and histopathology has not been described in detail, and is nearly unknown in the environment of diagnostic pathologists. In this article we want to explain the main theoretical aspects and the derivatives of sampling which are performed in routine tissue – based diagnostics. The derived formulas will allow interested pathologists or scientists to search for applications that can diminish the sampling time in virtual slides.

### Basic aspects of sampling in digitized histological slides (virtual slides)

Surgical pathology is a medical discipline that "extracts" information from human tissue and classifies the information in distinct terms that are called diagnoses. The common performance is to screen an organ or a tissue section for those spaces or areas that contain the most significant information, and try to classify this information seen in the specific field of view. Thus, tissue – based diagnosis is based upon a procedure to search for small samples that allow to derive information that is valid for the whole (or even patient). In other words, an appropriate sampling procedure is a precondition to evaluate accurate and reproducible diagnoses [2,4,11-15]. Therefore, a detailed definition and accurate description of the sampling method is a necessity if we want to further evaluate the diagnostic algorithms. This statement induces the definition of sampling as follows: Sampling is a method to derive information from a limited (small) compartment of a large (even unlimited) system that is valid for the entire (basic) system. The system can be a space, a function or set of functions, a body, an organ, a slide, or a DNA sequence.

The definition includes the term information, which has again to be defined: Information is a property that is exchanged between a sender and a receiver. Information is a property that can be understood by, and allows the receiver to react in an adequate, i.e., predictive manner. This definition of sampling includes two different aspects, which depend upon each other:

1. the method of sampling, and

2. the aim of the sampling procedure, i.e., which information should be extracted.

Different aims can require different methods of sampling, or at least different parameters of the same algorithm. The inclusion of an "aim" or "goal" to be assessed introduces the calculation of efficiency, or a cost/benefit estimation.

The most frequently used sampling goals are

➢ search for localization of specific items within the basic space, with the knowledge or assumption, that the space under consideration contains such items, and

➢ search for presence of specific items (tumour cells, ores, lobster, etc.), where the exact localisation of these items is of minor interest (for example localization of tumour cells in a cytological smear).

The prepositions to apply an adequate sampling procedure in tissue – based diagnosis include that number and size of the samples are limited. In addition, the detectable information has to be known. This information commonly depends upon additional (external) factors, and can be translated into diagnostic features that allow the detection and identification of a probe within the sampling space. These features can depend upon the size of the probes, their number, and their position within the collective, or even within the sampling space.

Let us assume that the final goal of our sampling method is the extraction of information from the entire space, and the classification of this information into a diagnosis. The diagnostic process can be separated into two different procedures:

I) the evaluation of a diagnostic significance of a certain object or "basic unit" which is the probability that the object can be grouped into a certain diagnosis, and

II) the probability to detect these basic units within the entire space.

**Figure 1:**
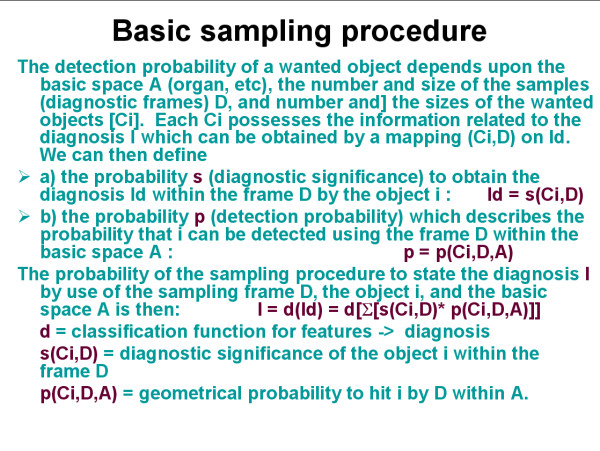
**Survey of sampling algorithm**.

**Figure 2:**
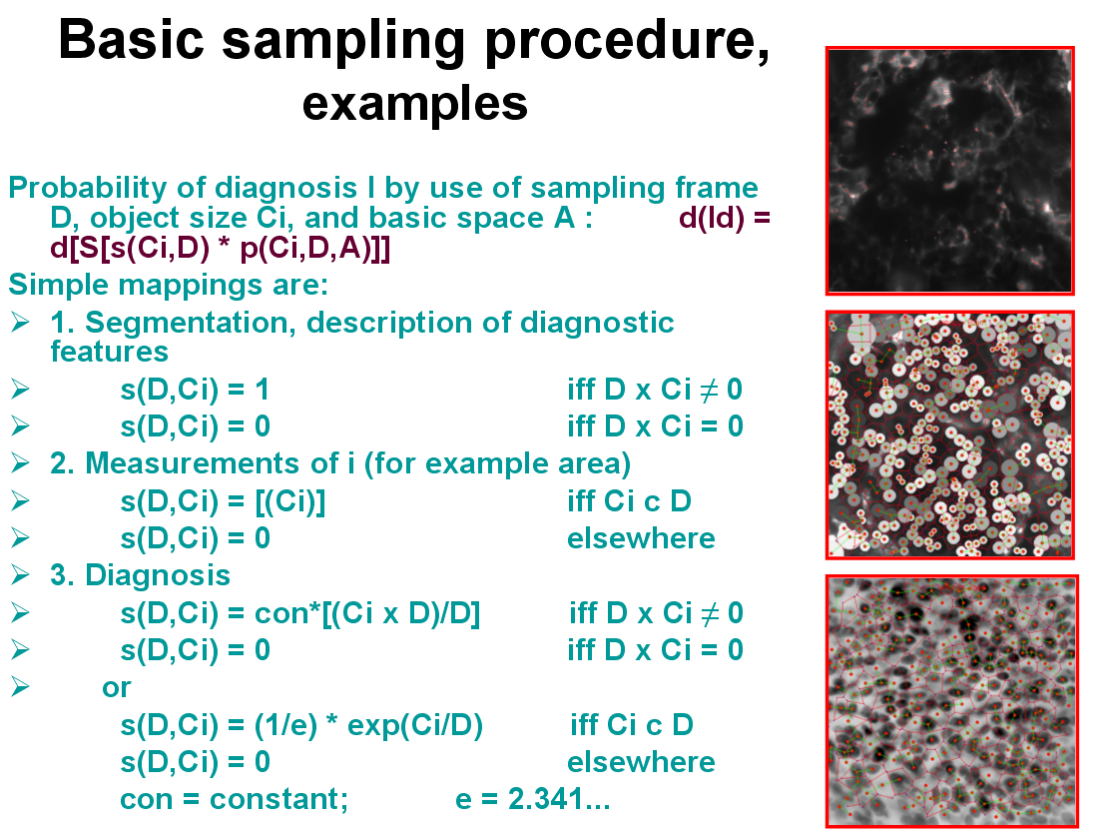
**Examples of different sampling procedures**.

**Figure 3:**
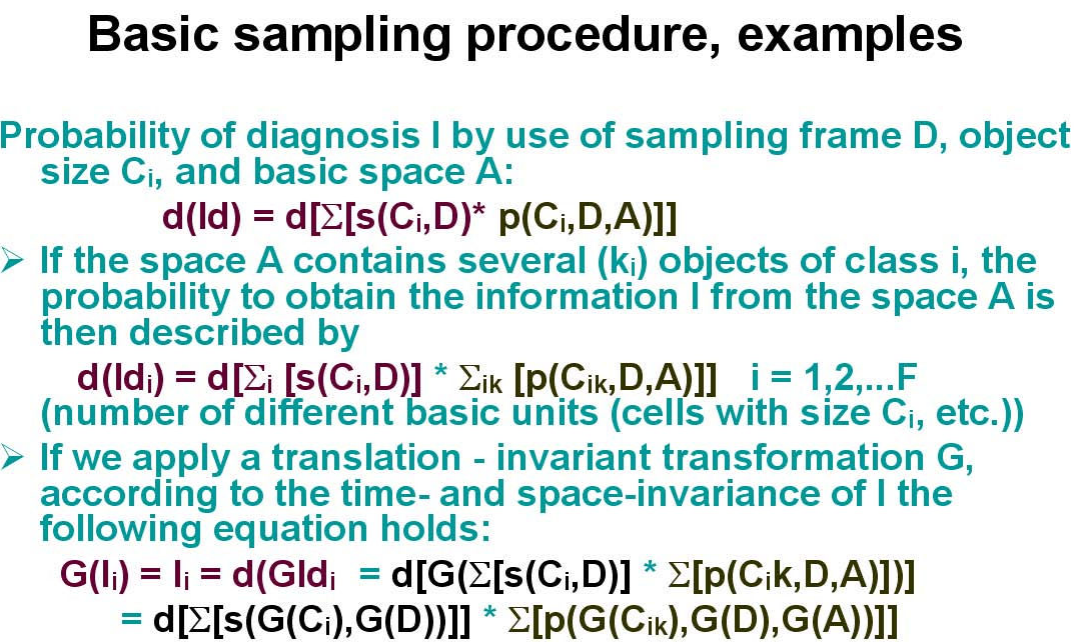
**Examples of probability calculations in various sampling procedures**.

**Figure 4:**
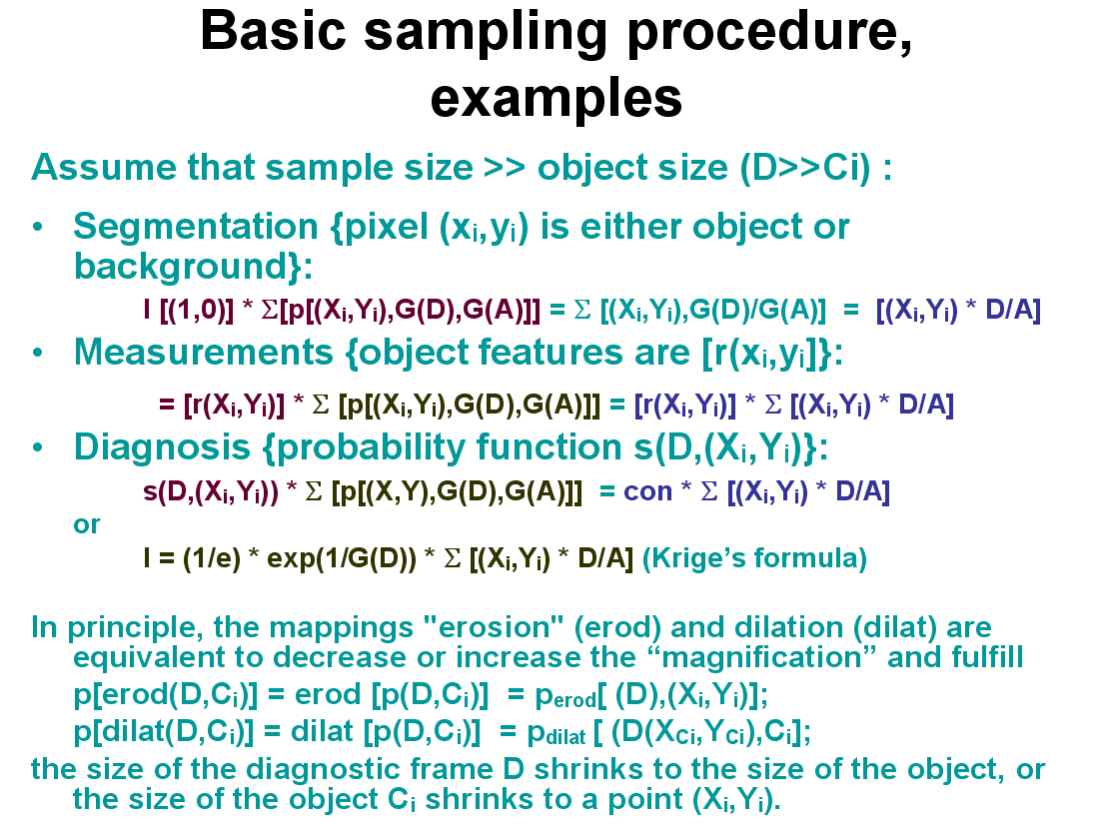
**Examples of sampling procedures in segmentation and diagnosis classification**.

The detection probability of a wanted object depends then upon the size of the basic space A (filed of view, organ, nucleus, etc), the number and size of the samples (diagnostic frames) D, and number and the sizes of the detected objects [Ci], as demonstrated in figure 1. Each detected object Ci possesses a certain probability to contribute to the diagnosis I which can be obtained by a mapping (Ci, D) on I_D_. I_D _is the probability to state the diagnosis I within the frame D by the object i, or ID = s(Ci, D). Basic examples are given in figure 2, figure 3, and in figure 4. In principle, the differentiation of the mapping of (Ci, D) on I_D _into the two different procedures, namely a) the detection (geometric significance) and into the diagnostic contribution reflects to the applied segmentation algorithms. These distinguish between areas (pixels) that contributed to the object and those that do not. Measurements of objects can only be done if the object is completely covered by the sample frame. The spatial selection of the samples can either be performed randomly, or dependent upon the localization of already segmented objects (stratified sampling). Stratified sampling is based upon the general law of self organization, i.e., similar biological systems tend to be localized in a neighbourhood relation. In other words, to detect a cancer cell is more likely in the neighbourhood of an already identified cancer cell than elsewhere. The function of diagnostic significance is often of exponential nature, and in light microscopy related to three different image properties, namely the texture, the object, and the object – associated structure [12,16-19].

### Sampling aims, applications and examples

Sampling is basically an information detection and transformation procedure, and thus undertaken to reach a certain final aim, for example to state a diagnosis, or to identify the presence or absence of certain objects. A time and space invariant translation of the sampling procedure can be assumed as long as we want to obtain reproducible results (figure 3). Such a translation permits a separation of the object detection likelihood from the diagnostic significance of the segmented objects, and allows us to compute both properties separately. Assuming a digitized image, each point (pixel x, y) within the basic image is either a presentation of an object or not. All object features can be reduced to a function that represents the object pixels in relation to sample and basic image size (figure 4). The introduction of an exponential diagnosis function then gives us the well known formula of Krige, which is commonly used to detect ores, oil fields, or underground water reservations [20]. Furthermore, the application of specific mappings (dilation, erosion) permits us to "increase the magnification of an object within its sampling frame, or to define the center of gravity in an object in order to compute the image structure. One will obtain a so – called order of structures if these procedures are repetitively applied to light microscopy [21].

### Random and stratified sampling procedures

**Figure 5:**
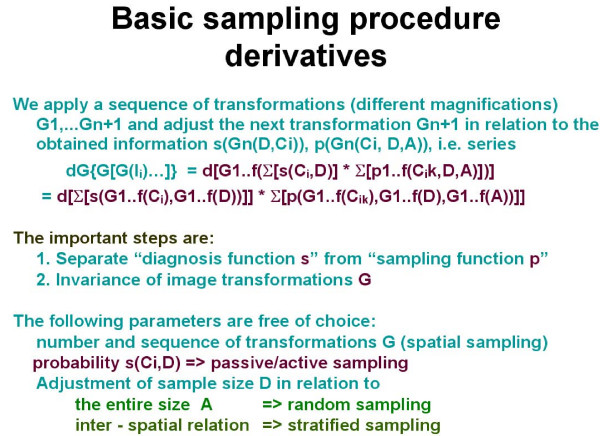
**Derivatives of basic sampling procedures in separating the diagnosis function s from the sampling function p**.

**Figure 6:**
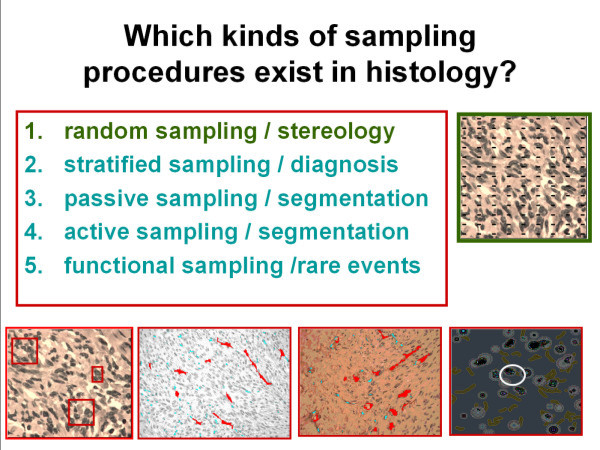
**Survey of different sampling procedures**.

The prerequisite of any (random, stratified) sampling is at least a binary image, i.e. a foreground defining the basic units and a background have to exist. Any sampling procedure can be performed either as random or stratified sampling figure 5 and figure 6. In addition, an active, passive, and a functional sampling can be distinguished.

Random sampling is the selection of biological meaningful units (nuclei, cells, proteins, etc.) at random. It is used to measure

➢ the frequency of the analyzed units in relation to each other or to the basic space (structure);

➢ certain features of the biological units in relation to the basic space (to further identify and classify the objects).

No information about the basic (reference) space is needed. The detection of biological meaningful units is then equivalent to the segmentation of the image and analysis of randomly chosen segmented elements. This procedure forms the basis of numerous investigations since the 1950s. It is commonly called stereology [22-26]. In principle, a grid consisting of regular lines (points) with identical length (and distance in between) is overlayed to the image, and the number of hits (intersections) is counted. From the number of intersections the volume – adjusted frequency, size, surface can be derived, independent from the orientation and shape of the elements. In a binary image the pixels (binary x, y points) can be used as a grid. Random chosen are the cutting angle (plane/volume), and the start point of the grid (pixel). Stratified are the selection of the grid (all pixels) and the count of intersections. Thus, any random sampling is provided by the start of the procedure, for example by random selection of the upper right position (x, y) coordinates of the sample space. From the relation *x/A *(number of hits *x/reference area A*) two-dimensional (and also three – dimensional) parameters can be derived. These include the area density (*Aa*), the volume density (*Vv*), the boundary density (*Ba*), the numerical density (*Na*), and the surface density (*Sv*). It should be noted, that this quite easily applied procedure permits the estimation of significant three – dimensional object features without any sophisticated three dimensional reconstruction [23,27,18,29].

Stratified sampling, in contrast, is provided by a specific selection of intersections (objects). Its objective is the detection of specific objects (of known features), and the measurement of features of known specific objects, or the estimation of objective-associated reference volumes, for example the density of proliferating cells related to distance from the nearest vessel [3,9,18,21]. Using again a grid as a measurement tool, the cutting angle (plane/volume) and the start point of the grid (pixel) are also randomly chosen. Stratified are the selection of the grid (all pixels), and the count of specific intersections (for example large cells) only. Thus: stratified sampling is provided by specific a selection of intersections (objects). A classic example is its application in cytology, i.e. to find the diagnosis-relevant cell (tumor cell) within a large number of "normal" cells. One could try to analyze

1. only those areas which contain features of (any) cell (gray value selection at low magnification)

2. within these areas only those cells which seem to be abnormal (gray value, size, moderate magnification)

3. within these cells those with abnormal nuclear size (DNA content), high magnification.

4. terminate the procedure once the diagnosis – significant information has been obtained

All other items are disregarded or neglected. The implementation of such an algorithm can speed up the time required "to screen a slide" significantly [5,12,30].

Stratified sampling requires some external knowledge in order to detect the biological meaningful events such as cancer cells. The image features of a cancer cell have to be known if one would like to detect this event by stratified sampling. The alternative algorithm would be to "sample" all cells, and start, if possible, a statistical analysis. This would then try to evaluate the rare events (supposing that cancer cells are rare to normal cells). Again, some external knowledge would be necessary. Obviously, this is related to the diagnosis function s(Ci, D).

Stratified sampling requires an accurate segmentation of objects with known features. Independent upon the actual segmentation procedure the sampling can be performed as active and passive sampling.

### Active and passive sampling

Any segmentation procedure has to accurately define the area of an object, which is equivalent to detect its boundary. Each pixel has to be distinguished either to belong to the object or not, which can be written: f(x, y, meaning) = [1,0], with f(x, y, object) = [1], and f(x, y, backgound) = [0] This approach is called passive sampling, as it discriminates the object area by a simple yes – no function [14]. In other words, passive sampling is provided by a constant relation between the objects and the grid (intersections). The intersection has the probability function p(i) = [1].

Active sampling is a different approach. It is provided by an objective-specific relation between the objects and the grid (intersections). The probability that a pixel belongs to an object ranges between [1,0]. The intersection has a probability function p(i, o), i.e., the probability to detect the pixels that belong to a certain object depends on the object itself and its neighborhood [20]. For example, a pixel displays a probability of 0.7 that it belongs to the object. This probability can increase or decrease dependent upon additional parameters, such as size, orientation, or shape of neighboring objects. Naturally, the probability value of 0.7 itself might be used to define whether it is an "object" – or a "background" pixel.

The probability function p(i, o) can be calculated if we separate p(i, o) in its two components: p(i, o) = gr(x, v) * af(gr, v).

gr(x, y) is the frequency distribution of different objects in the reference space v,

af(gr, v) is the detection probability in the space v.

If we assume that af(gr, v) = const in the reference space v, we can estimate p(i, o) by a set of measurements in different sample spaces and transform p(i, o) = [1] if gr(x, y) > const, and p(I, o) = [0] elsewhere.

**Figure 7:**
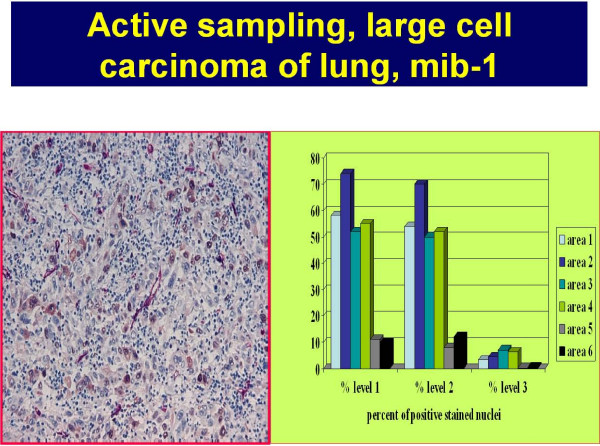
**Example of an active sampling to determine the discrimination threshold of a Mib-1 PAP stained undifferentiated large cell carcinoma (in association to the distance of the nearest vessel (proliferating nuclei are stained brown, vessels are stained red)**. The percentages of stained nuclei in relation to three different thresholds (level 1 – 3) are shown for six different sample areas. The discrimination threshold can be chosen between level 1 and level 2 without major error in contrast to level 3, which would result in wrong (too low) estimates.

Active sampling has been reported to be an effective method to correct the variation in immunohistochemistry staining, for example to compute the threshold of positive staining intensity [2]. The classic problem is: At which staining intensity (color level) can an "immunohistochemically analyzed cell" be grouped into the positive class? The active sampling attempt is to measure the relation positive/negative cells at different threshold levels in several randomly selected sample areas. We then select the discrimination threshold which results in (number of positive objects/number of negative objects) = const for all selected samples. A characteristic application is demonstrated in figure 7. It displays a Mib_1 stain for proliferating nuclei in a lung carcinoma, and the relative number of positive nuclei dependent upon three different thresholds measured in five different sample areas. Threshold number three is obviously too high, and the threshold number 1 too low. The threshold number two should be chosen as discriminating threshold, i.e., to calculate the relative number of proliferating nuclei. This algorithm has been successfully implemented in the automated immunohistochemistry measurement system (EAMUS™) [2,3,9,16,17].

### Functional sampling

The idea of functional sampling focuses on the interpretation of rare events [12,14,15,31-33]. The question arises: Do there exist certain rare cells within a cellular society (tissue) that possess a high functional power similar to catalysts in chemistry. If yes, how can they be identified? Therefore, functional sampling is defined as the search for a specific (key) function of rare biological objects within a different (majority) population. As the function to be analyzed might be unknown and we cannot observe the proposed function directly, we have to state the following prerequisites for proper analysis:

1. The specific object (cell) is rare within the basic population.

2. It has to possess regular neighborhood relations to objects of the basic population.

3. It has to be randomly distributed within the reference space.

The proposed algorithm tries to evaluate the distance properties between the rare events and the frequent events, and the general distribution of rare events within the reference space as follows:

1. We perform a random sampling of the specific (rare) object (*O*) within the basic population *Ni *(to estimating *O [Ni]*).

2. We perform a stratified sampling "around" each detected specific object (to estimating *Ni(0)*).

*3. If Ni(O) = constant *we can assume a specific function of the object (cell) within the basic population (for example cellular immune competence, functional activation of cells, etc.).

**Figure 8:**
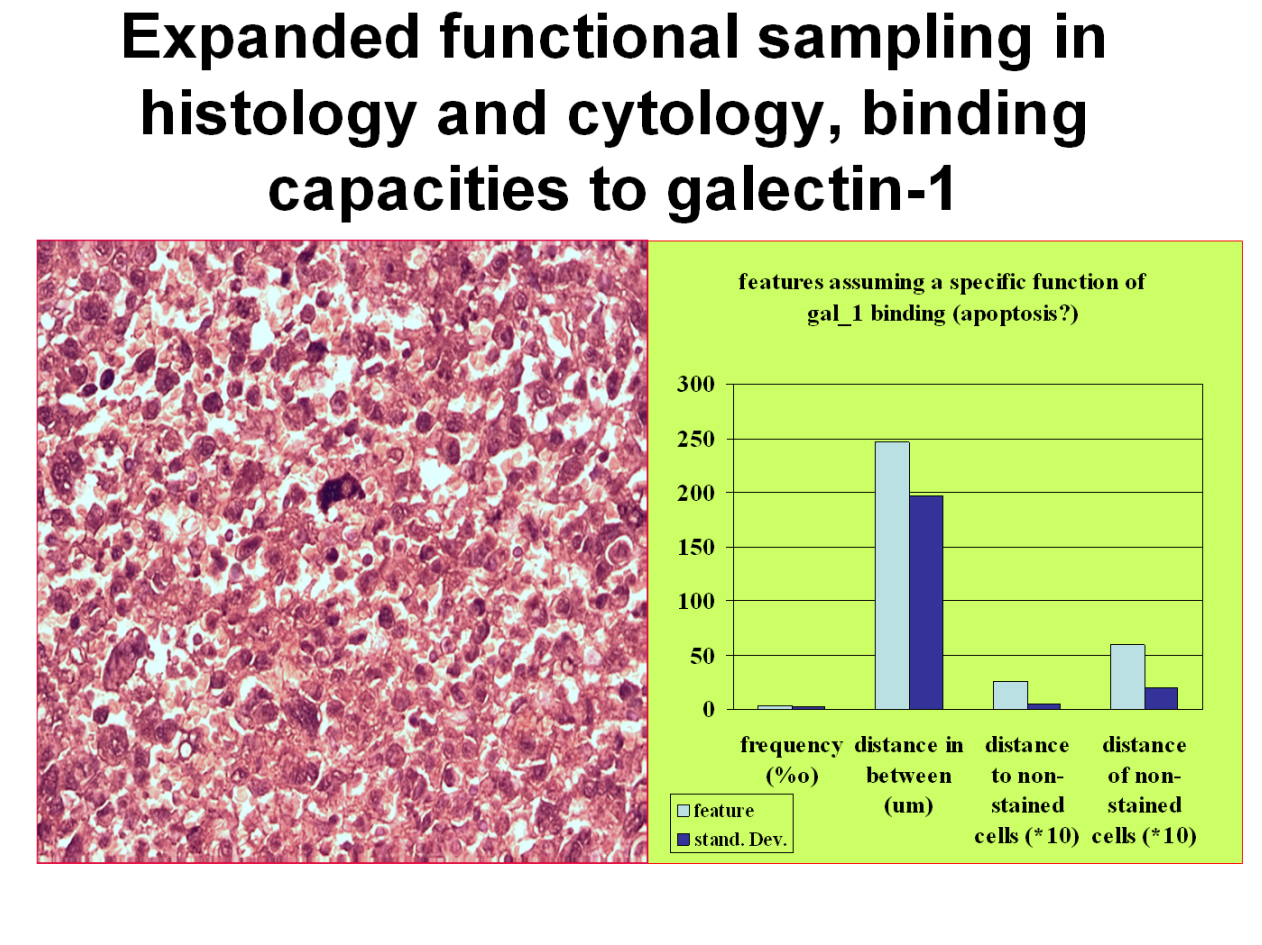
**Example of expanded functional sampling: A rare event (galectin-1 binding large cancer cell) within a large cell anaplastic lung carcinoma displays a regular distance to its nearest neighbouring cells and a random distribution within the whole slide**. In combination with biochemical data the result supports the idea that these cells might posses a "catalyst" function for progression of the total cancer cell population.

An example is shown in figure 8 which displays the binding capacities of labeled galectin-1 in an undifferentiated lung carcinoma. Only one large intensively stained cell is present in this sample area (marked by an arrow). Its frequency in all samples is < 5%, and the mean distance between these cells measures 245 ± 198 μm, i.e. these cells do not express a constant distance in between. The opposite, however, holds true for the distance between these large cells and their nearest (majority) cells as well as between the other cells within this tissue. From these figures we can derive, that the rare large cells probably posses a significant biological function for the whole tissue (according to biochemical investigations galectin-1 belongs to a family of galactoside binding proteins that has growth regulatory and immunomodulatory properties [21].

### Sampling efficiency

As we have defined sampling, it is a procedure that wants to describe space and time-related properties in surgical pathology, i.e. in tissue – based diagnosis. Such an investigation can be performed in different manners, which can be of different efficiencies. How can sampling efficiency be measured? Obviously, any sampling efficiency is closely related to the spatial distribution of the events searched for in the reference space. If the spatial distribution is known we can adjust the sampling procedure correspondingly. However, its spatial distribution is often not known, and we have to start with a random selection of certain compartments (samples). We can then measure the spatial distribution (frequency) p = s(N)/v, and the variation of p is the error E(p), which should be small in order to have an efficient sampling procedure. Usually, we can state that the reference space v >> s(Ne) (size of element e). The error E(p) can then be calculated according to

E(p) = Σ[E^2^(Ne) + E^2^(B(n)) + E^2^(Ne/sv)]^(1/2)^

with

E(Ne) = error of detecting an individual event (i.e., probability of identification/missing a tumor cell)

E(B(n)) = error of measuring all elements in the reference space (i.e., related to the biological variance of the tissue, dependent upon N)

E(Ne/v) = error of measuring the size of events e in relation to size of sampling space S (frequency of e in sample space sv).

We can derive the following statements from this formula:

1. We obtain the smallest sampling error if we select the reference volume as sample size, and if we are dealing with regular tissue (small biological variance).

2. The smaller the sample sizes in relation to the size of events, the bigger is the sampling error, as long as the error to segment (identify) per event is not increasing.

3. The sampling error is increasing if we choose different sizes of the samples.

## Discussion

To take and to analyze samples of a broad variety of tissues is a basic procedure in surgical pathology, or in tissue – based diagnosis. All diagnostic algorithms depend upon a correct and reliable sampling procedure, and extensive training in surgical pathology addresses to identify and sample those tissue compartments that probably contain the most significant information to classify the disease present [7,19,34-38]. The majority of investigations addresses to an optimum sampling procedure, for example. How many sentinel lymph nodes should be investigated in relation to the stage of breast cancer [29,31], or "optimizing sampling of tomato fruit for carotenoid content, or how to perform endometrial sampling in patients with trophoblastic disease after suction curettage [39,40]. In the early days of stereology several authors took attention on the sampling procedures, as the results of counting interceptions are closely associated to the nature of the used sampling method [22,23]. Recently, sampling has returned to the focus of investigations, especially in live imaging [41]. Most of the investigations try to optimize the sampling, which is equivalent to evaluate the "best" stratified sampling method.

In addition to medical applications, sampling plays a dominant role in geology, especially mining. In fact, Krige's sampling analysis can be considered to be the first approach to develop a "sampling theory" [12,20].

In this article we want to derive a scheme of sampling that permits a principle view of sampling, its different methods, and to calculate the efficiency of the used sampling method. In principle, two different algorithms exist, the random sampling and the stratified sampling [12,9]. Random sampling has to be performed, if no knowledge of the information searched for exists. It is the appropriate technique to measure features of biological units such as chromosomes, DNA fragments, nuclei, cells, vessels, etc. Its accuracy (error rate) can be predefined by number and size of the chosen samples in relation to the expected size of events and to the reference space. Its results can be implemented in additional classification algorithms, such as diagnostic procedures. The sampling can be terminated if a certain classification can be performed with a predefined accuracy, i.e, a diagnosis can be assessed with high certainty. The accurate measurement of events' features is a prerequisite, but not the aim of stratified sampling. Its implementation requires additional (external) information, and numerous investigations have been performed to "speed up" the procedure (or to make it more efficient) using spatial structures within the reference space. When an exponential event probability distribution is given, Krige's formula can be derived from stratified sampling.

In addition to the discussed principle differences between random and stratified sampling procedures, passive and active sampling plays a major role in image segmentation algorithms. The common principle of active sampling associates neighbourhood knowledge (i.e. knowledge derived from general external observations) to the object under investigation, for example to accurately define its boundaries [18]. Especially in measuring accurate thresholds for grading purposes in immunohistochemistry this approach has been proven to be successful [2]. A furthermore derived application is the functional sampling, which is again a stratified sampling in principle. This procedure can assist to investigate in the "biological importance" of rare events, which is widely not known to our experience.

In aggregate, a general theory of sampling is derived that possesses its applications in numerous, if not all natural sciences. They range from agriculture to mining, from aircraft maintenance to medicine. In surgical pathology it is of major importance that all diagnostic investigations start with appropriate sampling.

## Competing interests

The authors declare that they have no competing interests.

## Authors' contributions

KK and EV initialized the study and drafted the paper. HS, TG, JG and GK were involved in generating and evaluating the data and in the writing of the manuscript.
